# Malignant mesothelioma of the tunica vaginalis testis: a rare case and review of literature

**DOI:** 10.1186/s12885-020-6648-3

**Published:** 2020-02-27

**Authors:** Mingaile Drevinskaite, Ausvydas Patasius, Lukas Kevlicius, Ugnius Mickys, Giedre Smailyte

**Affiliations:** 1 Faculty of Medicine, Vilnius University, Vilnius, Lithuania; 2 Laboratory of Cancer Epidemiology, National Cancer Institute, Vilnius, Lithuania; 3 Department of Public Health, Institute of Health Sciences, Faculty of Medicine, Vilnius University, Vilnius, Lithuania; 4 National Center of Pathology, Affiliate of Vilnius University Hospital Santaros Clinics, Vilnius, Lithuania

**Keywords:** Malignant mesothelioma, Tunica vaginalis

## Abstract

**Background:**

Malignant mesothelioma of the tunica vaginalis is a rare tumour which comprises less than 1% of all mesotheliomas.

**Case presentation:**

69-years old patient with painful hard mass and hydrocele in the right scrotum to whom a right hydrocelectomy was performed. Any history of scrotal trauma or exposure to asbestos was not present. Excisional biopsy revealed a multinodular tumour with focal areas of necrosis and infiltrative growth. According to morphological and immunohistochemical findings, diagnosis of malignant biphasic mesothelioma of the tunica vaginalis testis was made. Two months after hydrocelectomy, right inguinal orchidectomy was performed. Post-surgical whole body CT scan revealed paraaortic and pararenal lymphadenopathy, likely to be metastatic. Adjuvant treatment with 6 cycles of cisplatin and pemetrexed was applied. After 3 cycles of chemotherapy, CT scan showed progression and the treatment was changed to gemcitabine 1 month after.

**Conclusions:**

Although malignant mesothelioma of the tunica vaginalis is a rare malignancy, it poses a diagnostic challenge which can mimic common inguinal or scrotal diseases such as hydrocele. Despite aggressive surgical procedures or adjuvant therapies, the prognosis remains poor.

## Background

Malignant mesothelioma of the tunica vaginalis testis (also known as paratesticular mesothelioma) is a rare tumour, comprising less than 1% of all mesotheliomas [1, 2]. Since the first record in 1957, only case reports and case series have been published, with less than 300 patients worldwide to date [3, 4]. The majority of these cases presented as an incidental finding during hydrocelectomy [4, 5]. Due to the rarity of this disease, epidemiology and risk factors are still unclear, and it is unknown whether asbestos exposure plays a role in the etiology of testicular mesothelioma [1, 6]. We present a case of malignant mesothelioma of the tunica vaginalis diagnosed after hydrocele surgery with a review of the literature.

## Case presentation

**Fig. 1:**
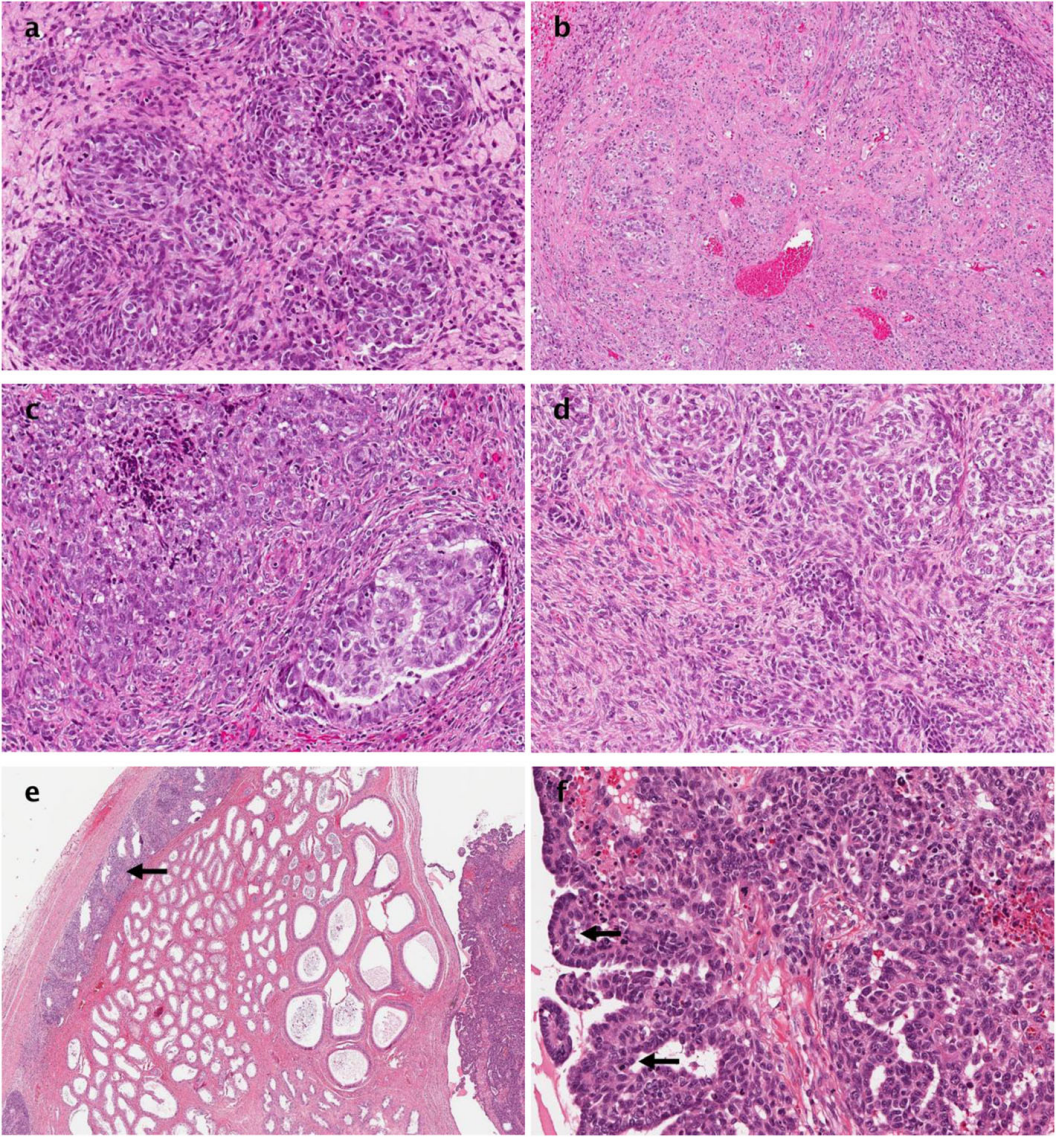
Histologic patterns, HE. (**a**) Nests of immature epithelioid cells, × 20; (**b**) Sarcomatoid areas mimicking desmoplastic response of the widely dispersed epithelial cells, × 10; (**c**) A highly cellular tumour with glomerular-like epithelial tufts projecting into cystic epithelium lined spaces, × 20; (**d**) An insidious right-to-left transition from epithelial to sarcomatous components, × 20; (**e**) Intracystic tumour with paratesticular spread, × 2; (**f**) The more-typical tubulopapillary pattern of mesothelioma, × 20

A 69-year-old man was referred to our hospital after the right hydrocelectomy when suspicious, non-homogeneous tissues were found intraoperatively. Ultrasound imaging and computed tomography (CT) scan were not performed before the surgery. On physical examination, right scrotum appeared to be edematous, hard and painful mass was evident with no palpable lymphadenopathy. Mentioned symptoms have been observed for a year. The patient denied any history of scrotal trauma or exposure to asbestos. 8 years ago he was diagnosed with prostate adenocarcinoma (Gleason 3 + 3 = 6) cT1cN0M0 stage II, the result of external beam radiotherapy (74 Grays / 37 fractions) treatment was radical. One year due to PSA (prostate specific antigen) relapse, there were androgen deprivation therapy prescribed. 9 months thereafter androgen deprivation therapy was discontinued due to low PSA rates. To the date of testicular mesothelioma diagnoses serum PSA level was 0.1 ng/ml. No other comorbidities were reported to this patient. Levels alpha-fetoprotein and beta human choriogonadotropin were not elevated. The excisional biopsy revealed a multinodular tumour with focal areas of necrosis and infiltrative growth. Mixed epithelial and spindle cell proliferation was present. The bulk of the epithelial component contained confluent cords and nests of monotonous, ovoid to polygonal cells with pale or eosinophilic cytoplasm (Fig. 1a). Focally, cell clusters were scattered in dense fibrous stroma or intermixed with moderately atypical spindle cells (Fig. 1b). Few glomeruloid structures were noted (Fig. 1c). There was no distinct transition from epithelioid to sarcoma-like areas (Fig. 1d). Diffuse immunoreactivity for cytokeratin AE1/AE3, calretinin, WT1, vimentin and CD10 was seen, with focal positivity for desmin in the epithelial component and smooth muscle actin in the sarcomatous part. CK5, EMA, PSA, NKX3.1, CK7, CK20, BerEp4, TTF-1, Cdx2, Pax8, SALL4, alpha-inhibin, ER, AR, LCA, myogenin were negative. According to morphological and immunohistochemical findings, diagnosis of malignant biphasic mesothelioma of the tunica vaginalis testis was made.

**Fig. 2:**
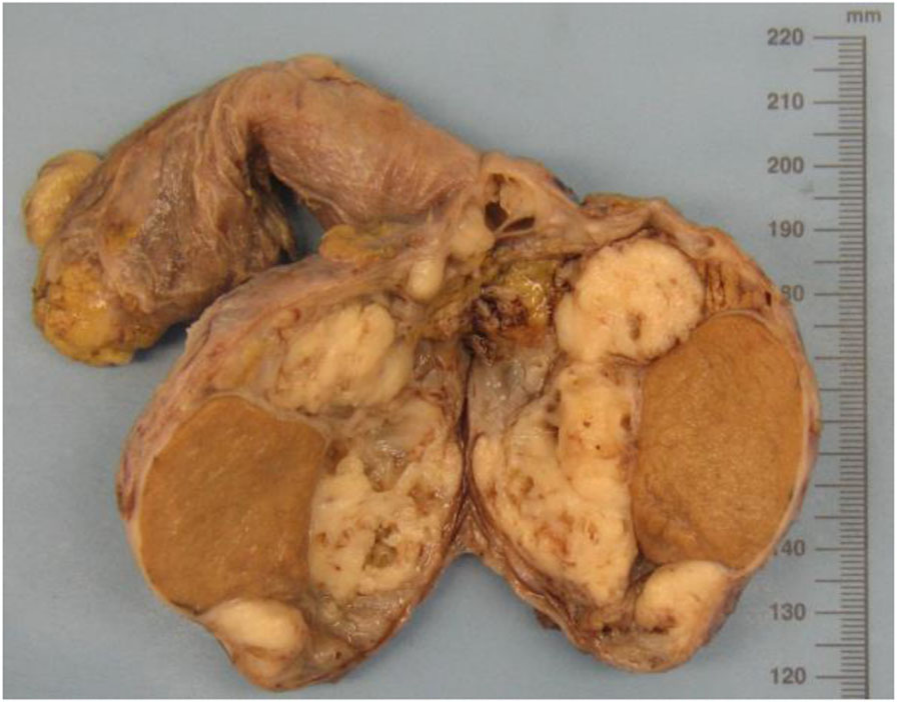
Resected specimen shows 3 cm × 7 cm nodular masses encasing the compressed testicular tissue

**Fig. 3:**
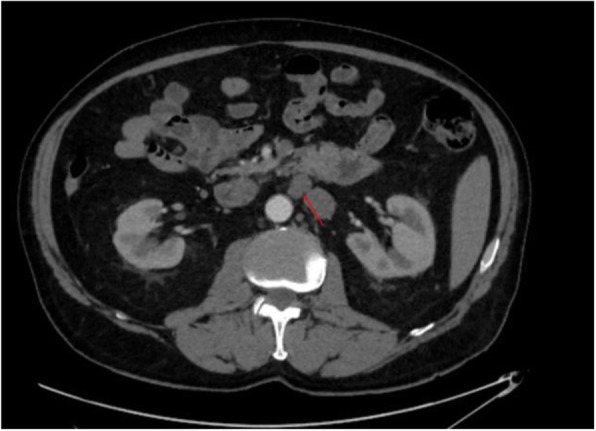
Enlarged to 21 × 15 mm infrarenal paraaortic masses in computed tomography axial view

**Fig. 4:**
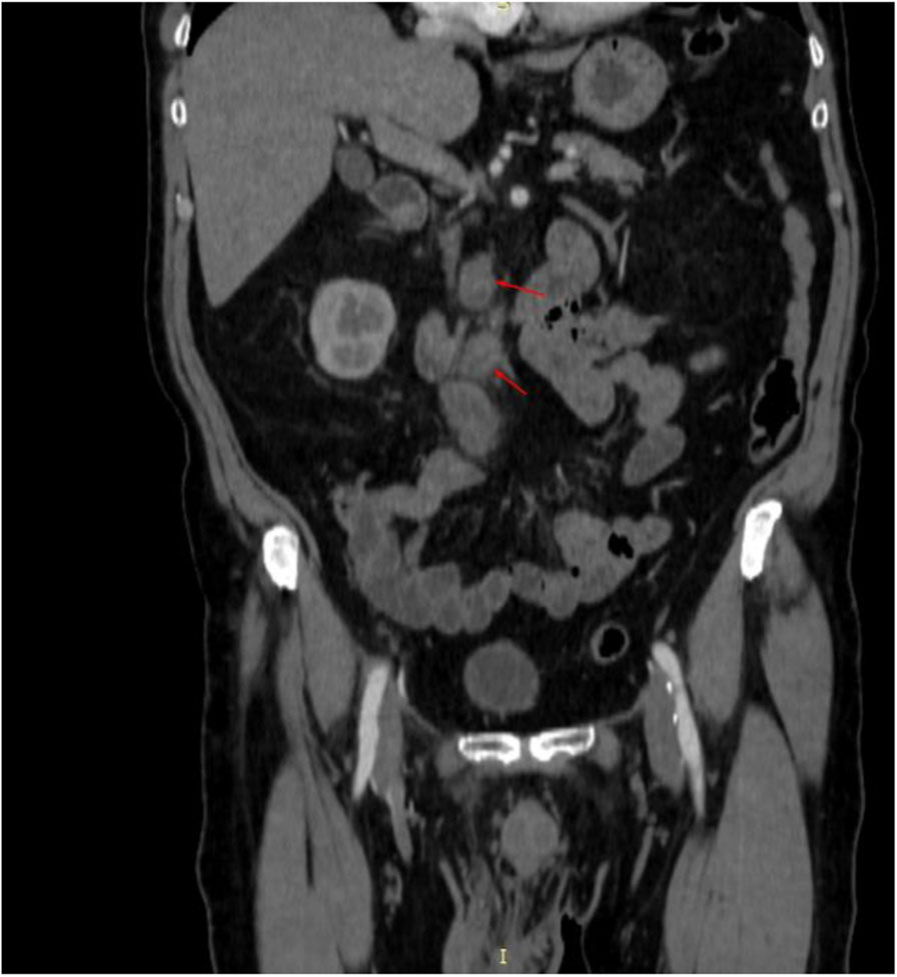
Infrarenal paraaortic masses enlarged to 21 × 25 mm, next to aortic bifurcation masses enlarged to 15 mm in computed tomography coronal view

**Fig. 5:**
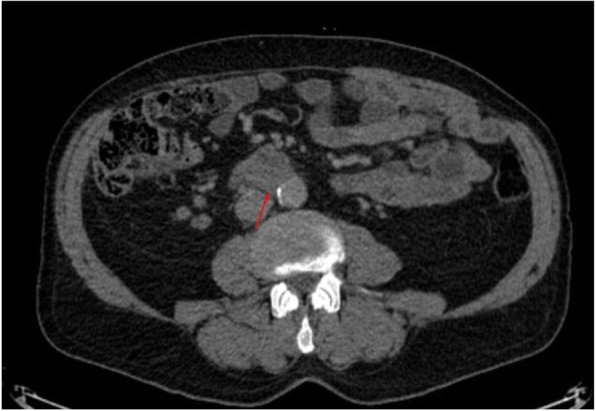
Radiological progression of disease with paraaortic mass, enlarged to 32 × 28 mm in computed tomography axial view

**Fig. 6:**
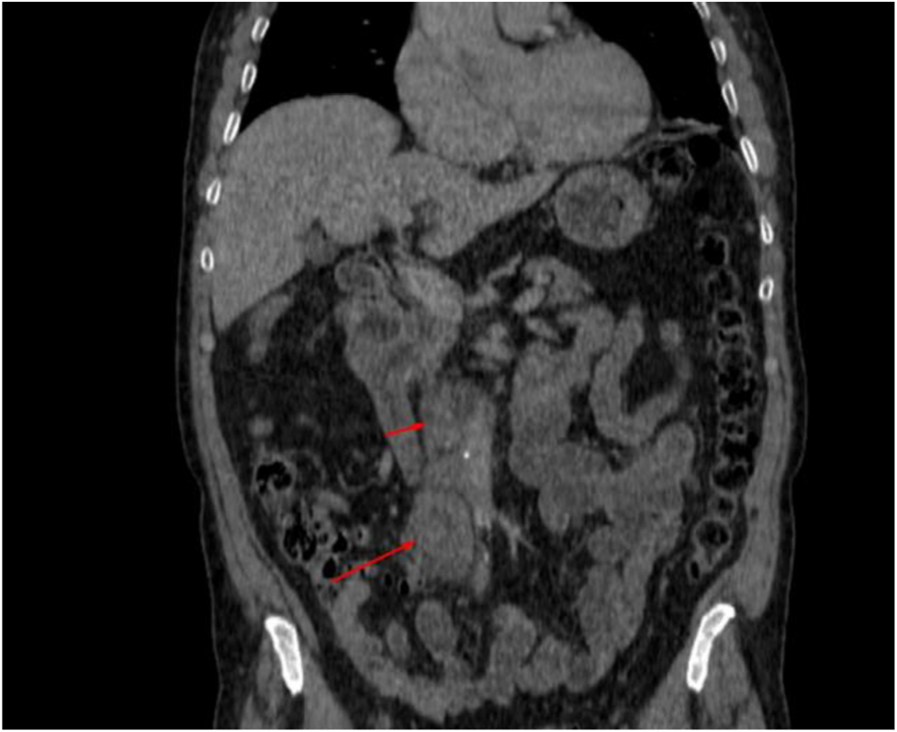
Paraaortic mass enlarged to 32 × 28 mm and mass next to aortic bifurcation, enlarged to 31 mm

Two months after hydrocelectomy, right inguinal orchidectomy was performed. Paratesticular space was largely replaced with yellow-white nodular masses with cystic spaces (Fig. 2). The subsequent histological examination confirmed the diagnosis (Fig. 1e and f). Post-surgical whole body CT scan revealed paraaortic and pararenal lymphadenopathy, likely to be metastatic (Figs. 3 and 4). The multidisciplinary team indicated adjuvant treatment with 6 cycles of cisplatin and pemetrexed. Three months after initiation of systemic treatment (3 cycles of cisplatin and pemetrexed), CT scan showed progression (Figs. 5 and 6) and the treatment was changed to gemcitabine 1 month after. Seven months after initiation of systemic treatment, patients status deteriorated significantly (ECOG – 3), accompanied by unmanageable pain and patient was transferred to palliative care centre. The patient died 9 months after the diagnosis.

## Discussion and conclusions

Malignant mesotheliomas are uncommon tumours, which can develop from the coelomic epithelium at the pleura, peritoneum, pericardium and tunica vaginalis testis. Mesothelioma of the tunica vaginalis testis represent only 0.3–5% of all mesothelial neoplasms [7]. According to Cancer Registry data in Lithuania 10–15 of mesotheliomas are reported each year. Since 1978 there was one patient with pericardial mesothelioma and none had mesothelioma of the tunica vaginalis testis to this date.

Testicular mesotheliomas usually occur between 55 and 75 years [5]. According to a population study in the United States, there were 52 testicular mesothelioma cases of 7101 malignant mesotheliomas diagnosed among males between 1973 and 2013 [1]. The incidence rate increased with age as males over the age of 80 had an 18.6 times higher rates than males under the age of 50 [1].

Exposure to asbestos is a major risk factor for the development of pleural and peritoneal mesotheliomas. However, asbestos link to tunica vaginalis tumours is not well elucidated [1, 7, 8]. In the literature review by Mezei et al., among the 89 reported testicular mesotheliomas the possibility of asbestos exposure was considered for 50 patients (56%) and confirmed or assumed for 30% [1]. In a general review of 223 cases, Bisceglia et al. found an association with asbestos exposure in only 30–40% [9]. The registry study of Marinaccio et al. provided no direct evidence that testicular mesothelioma was associated with asbestos exposure. The authors report that there was only one case in the highest risk industries such as asbestos cement, national defence, shipbuilding and railway industries [2]. Studies of asbestos exposed occupational cohorts reported no cases of malignant testicular mesothelioma as well [8]. The patient in our report is a 69-years-old man with no known history of asbestos exposure. Published literature marks an inconsistent relationship with asbestos and alone do not allow for any definite causal association to be made.

Other suspected risk factors for the disease include trauma, herniorrhaphy, long term hydrocele or spermatocele, long-term epididymitis, orchitis or other inguinal inflammation [1, 3, 4, 7, 10–14]. The mechanism by which chronic serosal inflammation contributes to the development of mesotheliomas are not known, although it has been suggested that they may be mediated via continuous production of interleukin-6 [15]. The patient in our case report denied any inguinal trauma or inflammation, or other possible risk factors, however, long-term hydrocele for a year and history of prostate cancer were evident. The first known study considering long-standing hydrocele and the risk of developing testicular mesothelioma was published in 2001 [14]. Unfortunately, there are no analytic epidemiologic studies done to prove this relation.

Therapeutic radiation for other malignancies is a well-established risk factor for pleural and peritoneal mesotheliomas [16, 17]. The etiologic link between ionizing radiation and malignant mesothelioma of the tunica vaginalis is plausible, due to radiation therapy causing multiple types of cancer [1, 7, 17]. It should be noted that our patient received an external beam radiotherapy treatment 8 years ago when he was diagnosed with prostate adenocarcinoma. Similar report was published in 2000 by Ferri et al. Their patient was diagnosed with prostate adenocarcinoma, treated surgicaly followed by radiation therapy. After 3 years, a pleural seroma, a cutaneous mass and testicular nodule were observed and cytological examination showed endothelial cells. Scrotal orchidectomy was performed, and further examination confirmed malignant mesothelioma of the tunica vaginalis testis [18]. Yet, there are no studies done, except a few reports, to prove the relation between radiation of prostate adenocarcinoma and testicular mesothelioma [1].

The rarity of testicular mesothelioma poses challenges to its diagnosis and that is rarely achieved preoperatively. Main symptoms are nonspecific and patients usually present with enlargement of the scrotum, recurrent hydrocele and palpable scrotal mass [19]. Commonly suspected clinical diagnoses include epididymitis, scrotal or inguinal hernia and spermatocele [9]. Ultrasonography (US) is non-invasive, simple and 90% accurate method when used to detect testicular tumours [20]. However, only case series describe imaging features of testicular mesotheliomas. Bertolotto et al. concluded that the most common US finding in the majority (5/10) of their patients was hydrocele with hypervascular parietal vegetations, while three patients had non-specific solid masses without hydrocele [21]. In our case, US and other imaging methods were not evaluated. Even though preoperative diagnosis could potentially lead to more aggressive surgical approaches and better survival, most tumours are identified intraoperatively on the basis of hemorrhagic hydrocele fluid, white – yellow nodules or papillary excrescences of the tunica vaginalis testis, followed by pathologic examination [2, 19].

In typical cases tunica vaginalis mesotheliomas resemble their pleural and peritoneal counterparts [22]. Pure epithelioid and biphasic histologic subtypes comprise 60–75% and 20–40% of all testicular mesotheliomas respectively and only few cases of pure sarcomatoid differentiation were identified [23, 24]. The tumour usually show papillary, tubulopapillary or well-differentiated solid growth with or without spindle cell proliferation [22, 24]. However, the wide morphologic diversity is a well-recognized feature [25]. The most commonly cited differential diagnosis include carcinoma of the rete testis, ovarian epithelial-type tumours of testis and paratestis, secondary adenocarcinomas, adenomatoid tumour and pleomorphic sarcomas [22, 24, 26, 27]. In our case, the pre-operative biopsy specimen lacked common features of mesothelioma, while the sex cord-like appearance (i.e. uniform low-grade epithelioid cells organized in sheets, cords, nests with blurred epithelial-stromal interface) was striking. Sex cord-stromal tumours are rarely mentioned as potential mesothelioma mimics and its histological pattern in the testicular region is prone to multiple diagnostic errors. This points out that low-volume paratesticular biopsies should be interpreted with caution and a wider immunohistochemical panel could be of major diagnostic value.

There is scarce evidence regarding treatment of this disease. Therapeutic principles are frequently extrapolated from management of pleural or peritoneal mesotheliomas and may feature a combination of surgery, radiation therapy and chemotherapy [28]. In our case, radical inguinal orchidectomy was performed after diagnosis, with radical intent. Although, presence of distant metastasis was unknown. As inguinal lymph nodes were not palpable, lymphadenectomy was not indicated [12]. Permetrexed with cisplatin, which have had a proven efficacy in pleural mesothelioma, are most often used in cases with unfavorable prognosis [29, 30]. Being diagnosed with metastatic disease, our patient received 4 cycles of cisplatin and pemetrexed. However, systemic treatment appered to be uneffective and gemcitabine was prescribed as 2nd line treatment. Gemcitabine is a valid options for patients, who do not respond well to platinum-based chemotherapy drugs [31]. Higher tumour stage (tumour, greater or equal to four centimeters, nodal involvement and metastasis) and biphasic histologic subtype are associated with significantly worse outcomes of testicular mesotheliomas [23]. More than 50% of patients develop local or distant recurrence with more than 60% recurrences within the first 2 years (5,19,29). Long-term follow-up should be assured, as one of case from study in The Johns Hopkins Hospital reported a reccurence after 15 years [6]. Of the total of 113 patients analyzed by Nazemi et al., the reported 5-year and 10-year overall survival was 49 and 33% respectively [23].

In summary, we present a case of a patient with testicular mesothelioma of tunica vaginalis. The rarity of testicular mesothelioma poses challenges to its etiology research, diagnosis and treatment. Diagnosis of testicular mesotheliomas is challenging, as the tumour lacks specific clinical and radiologic features, and the reported sex cord-like pattern proves its histological diversity. Despite aggressive surgical procedures or extratesticular mesothelioma-based adjuvant therapies, the prognosis remains poor.

## Data Availability

All data generated or analyzed during this study are included in this published article.
